# Thermophysiology and Locomotor Performance of the Andean Lizard *Phymaturus williamsi*: Vulnerable to Rising Temperatures?

**DOI:** 10.3390/biology15090729

**Published:** 2026-05-05

**Authors:** Ana E. Victorica Erostarbe, Franco Valdez Ovallez, José M. Sánchez, Yamila Méndez Osorio, Rodrigo Gómez Alés

**Affiliations:** 1 Gabinete DIBIOVA (Diversidad y Biología de Vertebrados del Árido), Departamento de Biología, Facultad de Ciencias Exactas, Físicas y Naturales, Universidad Nacional de San Juan, Av. Ignacio de la Roza 590 (O), Complejo Universitario “Islas Malvinas”, Rivadavia, San Juan J5402DCS, Argentina; francovaldez408@gmail.com (F.V.O.); mendezyami20@gmail.com (Y.M.O.); 2 CONICET (Consejo Nacional de Investigaciones Científicas y Técnicas), Av. Ignacio de la Roza 590 (O), Complejo Universitario “Islas Malvinas”, Rivadavia, San Juan J5402DCS, Argentina; 3 Instituto de Diversidad y Ecología Animal (IDEA), Consejo Nacional de Investigaciones Científicas y Técnicas (CONICET), Rondeau 798 (Parque de la Biodiversidad), Capital, Córdoba 5000, Argentina; unc.jose@gmail.com

**Keywords:** global warming, ectotherms, thermoregulation, thermal safety margin, Liolaemidae

## Abstract

Ambient temperature strongly influences the physiology and performance of ectotherms, making it essential to understand their responses to thermal variation under climate change. We evaluated locomotor performance and thermal vulnerability in a population of *Phymaturus williamsi* from the Argentine Andes. Field measurements included body (*T_b_*) and operative temperatures (*T_e_*), while laboratory analyses assessed preferred temperature (*T_pref_*), thermoregulatory effectiveness, critical thermal limits, and thermal sensitivity of locomotion (endurance, sprint, and long runs). *P. williamsi* exhibited a preferred temperature higher than field body temperatures, together with high thermoregulatory effectiveness (*E* = 0.83) and a broad thermal tolerance range (*TT* = 35.41 °C). Optimal temperatures for locomotor performance were lower than *T_pref_*, providing no support for the thermal coadaptation hypothesis. Despite low environmental thermal quality, individuals maintained body temperatures close to performance optima through effective behavioral thermoregulation. Thermal vulnerability indices indicated relatively high tolerance to projected warming scenarios. However, our results suggest that vulnerability to climate change is constrained more by the availability of suitable thermal microhabitats than by intrinsic physiological limits. We conclude that *P. williamsi* is a eurythermic and effective thermoregulator with thermally sensitive locomotor performance, whose persistence will depend on habitat structure and thermal heterogeneity.

## Simple Summary

Some animals, such as lizards, rely on ambient temperature to function properly, so understanding how they respond to changes in temperature is key to anticipating the effects of climate change. In this study, we analyzed how a species of lizard from the Argentine Andes (*Phymaturus williamsi*) regulates its temperature and how this influences its ability to move. We measured its body temperature in the wild and the temperatures available in the environment. In the laboratory, we assessed which body temperatures they prefer, how much they tolerate, and how their movement performance changes at different body temperatures. We found that these lizards prefer higher body temperatures than those they experience in their environment, but they still manage to regulate themselves efficiently using their behavior. We also observed that they can tolerate a wide range of temperatures and that their best movement performance occurs at lower body temperatures than they prefer. Taken together, these results indicate that the species has a good capacity to cope with thermal changes. This is important because it suggests that it could withstand the effects of global warming in its natural environment relatively well.

## 1. Introduction

Numerous studies have attempted to predict the vulnerability of organisms to global warming, taking into account future projections [1,2,3,4,5,6,7]. For example, Sinervo et al. [3] predicted that 39% of lizard populations worldwide and 20% of lizard species globally could become extinct by 2080, assuming limited plastic and evolutionary responses to a warming world and continued inaction to reduce greenhouse gas emissions. Ectotherms are particularly vulnerable to changes in environmental thermal conditions because they rely on external heat sources to regulate their body temperature [8,9,10]. In this context, understanding how these organisms cope with thermal variation is essential to predict their ecological and evolutionary responses, as well as to assess their risk of extinction [2,11].

Lizards rely on behavioral thermoregulation, such as basking and microhabitat selection, to attain optimal body temperatures. However, these strategies may become insufficient when maximum environmental temperatures exceed physiological performance limits [12,13]. For these organisms, the physiological benefits of thermoregulation inevitably entail costs, including increased energetic expenditure associated with moving between warm and cool microhabitats and greater exposure to predators [14]. For example, species inhabiting desert environments must balance overheating avoidance with water conservation through microhabitat selection and temporal shifts in activity [15,16,17]. Moreover, time allocated to thermoregulation cannot be devoted to other essential activities such as reproduction, foraging, social interactions, and territorial defense [14,18,19]. Consequently, the ability of species to buffer the effects of warming through behavioral adjustments depends on the availability of suitable thermal microhabitats, habitat heterogeneity, and inherent physiological constraints [6,20,21,22]. In particular, endemic species—especially those inhabiting cold mountainous regions—are considered to face a higher extinction risk than widely distributed species [23,24]. In this context, global warming represents a significant threat to the biodiversity of these reptiles, particularly for species with restricted distributions and specific thermal requirements [3,10]. Experimental and field studies further show that increasing temperatures are already limiting daily activity and promoting greater use of thermal refuges in lizards from temperate regions [20,25,26].

A widely used approach to evaluate the ecological effects of increasing temperatures is to examine how body temperature influences individual performance, including traits such as locomotion [10,27,28,29,30]. In reptiles, locomotion represents a key temperature-dependent functional trait because it underlies numerous ecologically relevant activities, such as prey capture, reproductive behavior, and predator avoidance [31]. The relationship between temperature and locomotor performance is commonly described by a thermal performance curve (TPC), which allows the estimation of both the optimal temperature for maximal performance (*T_o_*) and the breadth of thermal tolerance, for example *B*_80_ [27,32]. Within this framework, the thermal coadaptation hypothesis predicts that the optimal temperature (*T_ₒ_*) of thermal performance curves should coevolve with preferred body temperature (*T_pref_*), such that maximal performance occurs at body temperatures most frequently selected under natural conditions [9,33,34,35]. In addition, indices such as the thermal safety margin (TSM) and warming tolerance (WT) provide integrative metrics to estimate thermal vulnerability, reflecting the capacity of species to maintain physiological performance under increasing environmental temperatures [2,36].

The genus *Phymaturus* (Liolaemidae) currently comprises more than 52 recognized species distributed across the southwestern region of South America [37,38] and is considered a life-history conservative genus [39,40]. All species of *Phymaturus* are herbivorous, saxicolous, and viviparous, inhabiting cold and harsh environments of the Andes in Argentina and Chile [41,42,43]. In addition, species of *Phymaturus* exhibit low reproductive output: females have biennial reproductive cycles and small litter sizes [44,45,46]. Individuals also require between 7 and 9 years to reach sexual maturity [47,48,49]. These species are characterized by limited dispersal capacity and confinement to specialized habitats within restricted distribution ranges [50]. Such biological traits, together with their relatively conserved thermal physiology [51], make this genus particularly susceptible to the effects of climate change [3,11,40,52]. In particular, *Phymaturus williamsi* is a lizard endemic to Andean environments of the Calingasta Department in San Juan Province, Argentina [53]. Its restricted spatial use and reproductive adjustments associated with high-altitude environments may increase its sensitivity to climate change [19,44]. Despite this, and despite the overall vulnerability of the genus [50], *P. williamsi* is currently categorized as a species of “Least Concern” on the IUCN Red List, and therefore receives relatively low conservation priority [54].

Thermal vulnerability shows marked interspecific variation within the genus *Phymaturus*. For example, *Phymaturus calcogaster* exhibits broad thermal safety margins and high warming tolerance [55], whereas *Phymaturus palluma* displays high thermal sensitivity and potential reductions in its distribution under warming scenarios [18,40]. Likewise, comparative studies in the central Andes indicate that syntopic species of *Phymaturus* and *Liolaemus* differ in their thermoregulatory strategies, reflecting strong thermal niche segregation [32,56,57]. Recent assessments of *Phymaturus tenebrosus* and *Phymaturus verdugo* suggest that, although some species may benefit from moderate temperature increases, both intra- and interspecific variation may limit their capacity to adjust to climate change [27,38]. Nevertheless, the available information for *P. williamsi* remains scarce, and its potential physiological and ecological responses to global warming remain largely unexplored.

In this study, we propose that: (1) the preferred body temperature of *Phymaturus williamsi* (*T_pref_*) will be associated with its optimal locomotor performance temperature (*T_ₒ_*), consistent with the thermal coadaptation hypothesis [33,58]; (2) the species is able to maintain efficient thermoregulation, which may partially buffer the effects of environmental warming; and (3) the projected temperature increase of 3–3.5 °C [27,59] may reduce its thermal safety margins (TSM) and warming tolerance (WT), thereby increasing its physiological and ecological vulnerability. Although recent studies have characterized aspects of field thermoregulation in *P. williamsi*, including body temperatures and thermal preferences, its critical thermal limits, optimal locomotor performance temperature, and integrative indices of thermal vulnerability (TSM and WT) remain unknown. To evaluate whether *P. williamsi* is indeed vulnerable to increasing temperatures, we experimentally examined its thermal biology and locomotor performance in an Andean population from Calingasta (San Juan, Argentina). Specifically, we estimated field body temperatures, thermal preferences, critical thermal limits, optimal performance temperature (*T_ₒ_*), and thermoregulatory efficiency, and calculated TSM and WT. In doing so, we integrate thermal physiology and functional performance to determine the degree of exposure and sensitivity of this species to global warming, providing an integrative assessment of its potential vulnerability to climate change.

## 2. Materials and Methods

### 2.1. Fieldwork

Fieldwork was conducted at Quebrada La Puerta, Calingasta Department (32°31.31′ S; 69°42.02′ W; 2878 m a.s.l.), in the Andean Cordillera of San Juan Province, Argentina. This area belongs to the Puna phytogeographic province, located between 2300 and 3100 m a.s.l. [60], in the Andean highlands of Argentina on the western slope of the Iglesia–Calingasta–Uspallata Valley, which extends in a north–south direction. The climate is cold and dry, with a mean annual temperature below 8 °C, and is characterized by large daily temperature fluctuations, with nighttime temperatures dropping below 0 °C and daytime temperatures exceeding 30 °C [61,62].

We captured individuals of *Phymaturus williamsi* (*n* = 16) by hand and using nooses during October–November 2014. For each individual, we recorded field body temperature (*T_b_*) using thermocouples (TES TP-K01, TES Electrical Electronic Corp., Taipei, Taiwan; 1.62 mm diameter), snout–vent length (SVL; measured with a vernier caliper ± 0.02 mm), and body mass (BM; Pesola spring scale Pesola AG; Baar; Switzerland; ± 0.05 g). Sex was determined based on the presence of precloacal pores and hemipenal eversion in males, and by examining coloration and the presence of embryos or follicles in females. Considering the vulnerable conservation status and life-history traits of the genus *Phymaturus* [50], we aimed to work with the smallest possible sample size. Species in this genus are characterized by highly specialized biology, delayed sexual maturity, low reproductive output, and restricted distributions, which often result in small and sparse populations [50]. In addition, pregnant females were excluded from the study. Reproductive condition was determined by abdominal palpation following procedures previously applied in other *Phymaturus* species [27,63]. The experimental design was also structured to minimize time in captivity and reduce handling-induced stress in the studied individuals [27].

We recorded operative temperatures (*T_e_*, sensu Hertz et al. [64]), which represent the body temperatures that an individual would experience in the absence of behavioral thermoregulation, integrating both biotic and abiotic factors that influence *T_b_*. Operative temperatures were measured using six polyvinyl chloride (PVC) biophysical models placed in the microhabitats most commonly used by *P. williamsi* (bare soil, over and under rocks, crevices, and over and under bushes). Models were connected to data loggers (HOBO© ProV2; Onset Computer Corporation; Bourne, MA, USA), and temperatures were recorded every 5 min during the daily activity period (11:00–18:00 h) for four consecutive days. Sampling was conducted under typical seasonal weather conditions. This sampling design allowed us to characterize the operative thermal environment experienced by active individuals across representative microhabitats. *T_e_* values were obtained by averaging the readings from all models. For this study, we used models previously calibrated for *P. extrilidus*, as this species closely resembles *P. williamsi* in body size and morphology (e.g., similar snout–vent length and overall body shape) [64,65]. Importantly, these models have been previously validated for *Phymaturus* species of comparable size and shape, showing a strong correlation between model temperatures and live animal body temperatures (R = 0.98; [27,65,66]). Although using models calibrated for a different species may introduce some uncertainty, the high morphological similarity among species and prior validation suggests that any potential bias in *T_e_* estimates is likely minimal and does not affect the general interpretation of the thermal environment.

### 2.2. Laboratory Experiments

Captured lizards were individually transported in cloth bags to the laboratory at the Departamento de Biología, Universidad Nacional de San Juan (San Juan, Argentina). Upon arrival, each individual was housed separately in a terrarium (30 × 25 × 20 cm) to avoid contact between individuals and minimize stress. Each terrarium was equipped with a shelter, maintained under a natural photoperiod, and kept at a controlled ambient temperature of 24 °C to facilitate thermoregulation while preventing unintended acclimation [17,67]. Water was provided ad libitum, and terraria were cleaned regularly to ensure proper hygiene conditions. No signs of dehydration or abnormal behavior were observed. Lizards were not fed during the experimental period, following standard protocols for short-term physiological trials [28,58,68]. Individuals were maintained under these conditions for 8 days, a period considered sufficient to estimate thermal and locomotor performance traits.

All individuals underwent the following laboratory trials in the same sequence: preferred body temperature, locomotor performance (sprint and endurance), and critical thermal limits. We adhered to the guidelines for the management of amphibians and reptiles in field and laboratory research [69], as well as the regulations detailed in Argentine National Law no. 14346. This research was authorized by the Secretaría de Medio Ambiente, Dirección de Conservación y Áreas Protegidas, Province of San Juan (exp. no. 13004047, J.C.A.).

#### 2.2.1. Preferred Body Temperature

Thermoregulatory experiments were conducted 3–4 days after capture. Lizards were placed individually in terraria (150 × 45 × 80 cm). A thermal gradient (17–45 °C) was generated using infrared lamps of different wattages positioned at varying heights to create a linear gradient encompassing the natural thermal range observed in the field. Experiments were conducted during the species’ activity period (11:00–18:00 h), and body temperatures were recorded every 5 min for 3 h [66,70]. Body temperature was measured using ultrafine thermocouples (1 mm) inserted into the cloaca and secured with hypoallergenic adhesive tape. Thermocouples were connected to a TC-08 USB data logger (OMEGA^®^; Omega Engineering Inc., Stamford, CT, USA), allowing continuous monitoring without handling the animals. For each individual, we calculated the mean preferred body temperature (*T_pref_*). The lower (*T_setLo_*) and upper (*T_setUp_*) limits of the set-point range were estimated using the interquartile range (25–75%) of the recorded temperatures following Hertz et al. [64].

#### 2.2.2. Locomotor Performance

Sprint speed was measured using a linear racetrack (0.08 m wide × 1.2 m long) equipped with infrared sensors placed at 0.15 m intervals and connected to a computer that calculated speed across each segment. Two types of locomotor performance were evaluated: sprint runs (SR), time between the first and second sensors (0.15 m), simulating a predator-escape response; long runs (LR), time between the first and last sensors (1.05 m), representing sustained locomotor performance relevant to activities such as foraging and territorial defense (sensu Cabezas-Cartes et al. [71]).

Lizards were induced to run by gently tapping the base of the tail with light, continuous stimuli, taking care not to interfere with running performance [9,72]. Each lizard was tested at five randomly ordered temperatures within its thermal tolerance range (18, 21, 27, 32, and 35 °C). At each temperature, three consecutive runs were recorded, and the highest speed was taken as the maximum performance (*V_max_*) for both SR and LR. Only one temperature treatment was tested per day, with a 24-h rest period between trials. At the end of the experiment, the initial temperature treatment was repeated to assess potential fatigue effects [9,68]. Before each trial, lizards were allowed to equilibrate at the target temperature. From these trials, we calculated for each individual: individual maximum speed (*V_max,i_*), species-level maximum speed (*V_max_*), optimal temperature for performance (*T_o_*), thermal performance breadth (*B*_80_), defined as the range of temperatures at which performance was ≥80% of *V_max_* (Hertz et al. [73]; see statistical analyses).

Endurance capacity was assessed using a motorized treadmill following the methodology of Angilletta et al. [9] and Gómez Alés et al. [32]. Trials were conducted at three randomly assigned temperatures (27, 32, and 35 °C), selected to represent body temperatures experienced by the species in the field, including temperatures within the preferred range and values approaching the upper thermal conditions of the environment. Individuals were allowed to equilibrate at each test temperature for 30 min before trials. Lizards were then placed on the treadmill and gently stimulated on the hind legs to induce running. Endurance was quantified as the time (in seconds) each individual ran before reaching exhaustion at a constant speed of 0.15 m s^−1^. Individuals were considered fatigued when they were unable to right themselves after being placed on their backs [68,74].

#### 2.2.3. Critical Thermal Limits

We estimated minimum (*CT_min_*) and maximum (*CT_max_*) critical temperatures following the methodology of Gómez Alés et al. [75]. To determine *CT_min_*, individuals were placed separately in plastic terraria inside a refrigerator set at a constant temperature (−15 °C). Cooling rates were continuously monitored throughout the trials based on sequential body temperature measurements. Body temperature and righting response (assessed by placing the lizard in a supine position) were recorded at 60-s intervals. Handling was performed rapidly using gloves to minimize thermal disturbance. To determine *CT_max_*, individuals were placed in a cylindrical container (25 cm diameter × 35 cm height) with sterile sand as substrate. A 150-W incandescent bulb was positioned 50 cm above the sand surface. Body temperature was measured every 30 s, and individuals were gently inverted at the same intervals to assess the righting response. *CT_max_* was defined as the body temperature at which an individual lost the righting response when inverted. The onset of muscular spasms was used as an additional confirmatory cue [51,76]. We acknowledge that frequent inversion to assess the righting response may influence the accuracy of *CT_max_* estimates, and that the onset of muscular spasms does not necessarily coincide with the loss of the righting response [77,78]. However, both criteria were used here to provide a conservative and standardized estimate of upper thermal limits, consistent with previous studies in *Phymaturus* and other lizard taxa that rely on loss of righting response as an endpoint [27,28,32,55,79]. This approach allows for direct comparison with existing literature on thermal tolerance in the genus. In both *CT_min_* and *CT_max_* trials, individuals began at an initial body temperature of 20 °C, and heating and cooling rates were maintained at approximately 1 °C min^−1^ to ensure accurate estimation of critical thermal limits [17,51,72].

### 2.3. Thermoregulatory Efficiency and Thermal Vulnerability Indices

To estimate thermoregulatory efficiency (*E*) in the field, we calculated the average deviation between body temperature and the set-point range (*db* = deviation of *T_b_* from *T_set_*), and habitat thermal quality (*de* = deviation of operative temperature, *T_e_*, from *T_set_*) for each individual. Using the mean values of *db* and *de*, thermoregulatory efficiency was calculated following Hertz et al. [64]: *E* = 1 − (*db*/*de*). Values of E approaching 1 indicate highly effective thermoregulation, values near 0.5 indicate moderate thermoregulation, and values approaching 0 indicate thermoconformity [64].

We calculated the thermal safety margin sensu Deutsch et al. [2] (TSM_D_), which indicates how close an organism’s thermal optimum is to the current climatic temperature in its environment, as the difference between *T_o_* and mean *T_e_*. We also calculated the thermal safety margin (TSM) following Gómez Alés et al. [32], defined as the difference between *T_o_* and *CT_max_*. Finally, we estimated warming tolerance (WT), which represents how much environmental warming an ectotherm can tolerate before reaching lethal temperatures, calculated as the difference between *CT_max_* and mean *T_e_* [2,80]. Thus, TSM_D_ reflects the proximity of performance optima to current environmental temperatures, TSM represents the distance between optimal and upper critical limits, and WT quantifies the buffer between environmental temperatures and lethal thresholds.

### 2.4. Statistical Analyses

Thermal performance curves (TPCs) were estimated for each locomotor performance metric (long runs, sprint runs, and endurance). Because TPCs typically show an asymmetric shape, with a gradual increase in performance and a rapid decline above the optimal temperature, we modeled these relationships using Generalized Additive Mixed Models (GAMMs). GAMMs were fitted using the gamm function in the mgcv package in R [81]. Body temperature was included as a smoothing term, and individual identity was incorporated as a random effect to account for repeated measurements of the same individuals across temperatures. Snout–vent length (SVL) was included as a covariate to control for size-related variation in locomotor performance. To account for potential temporal autocorrelation among repeated measurements, alternative correlation structures were evaluated, including first-order autoregressive (corAR1), continuous-time autoregressive (corCAR1), and autoregressive moving average (corARMA) processes [82,83]. The best-fitting model for each locomotor trait was selected using the Akaike Information Criterion (AIC). From the fitted TPCs, we estimated the thermal optimum for performance (*T_o_*), defined as the body temperature at which locomotor performance was maximal, and the performance breadth (*B*_80_), defined as the temperature range at which performance was ≥80% of the maximum value. The critical thermal limits (*CT_min_* and *CT_max_*) were included to anchor the extremes of the TPCs [84,85], setting performance to zero at these boundaries.

To quantify the magnitude of differences between temperature treatments beyond statistical significance, we calculated standardized effect sizes (Cohen’s d) for all pairwise post hoc comparisons [86]. Following Cohen, [87], we interpreted d = 0.2 as a small effect, d = 0.5 as a medium effect, and d = 0.8 as a large effect. For repeated measures designs, Cohen’s d was approximated as d = t × √(2/n), where t is the t-ratio from the post hoc Tukey test and n is the sample size [86].

Relationships between physiological and morphological variables were evaluated using simple and multiple linear regressions. Comparisons between means were performed using paired or unpaired *t*-tests, and one-way repeated-measures ANOVA when appropriate, followed by post hoc tests. Normality and homoscedasticity assumptions were evaluated using Shapiro–Wilk and Kolmogorov–Smirnov tests. Results are presented as means ± standard error (SE) or medians when appropriate. Statistical significance was set at α = 0.05. All analyses were conducted in R version 3.6; R Foundation for Statistical Computing: Vienna, Austria [88].

## 3. Results

### 3.1. Thermal Biology

The mean body temperature (*T_b_*) of *P. williamsi* was 32.20 ± 3.24 °C (*n* = 16) and was not significantly related to snout–vent length (SVL) or body mass (multiple linear regression: F_2,14_ = 0.40, *p* > 0.05). Preferred body temperature (*T_pref_*) was significantly higher than *T_b_* (Wilcoxon signed-rank test: T = 16, *p* < 0.01). Summary statistics for *T_pref_* and set-point range are presented in Table 1.

**Figure 1:**
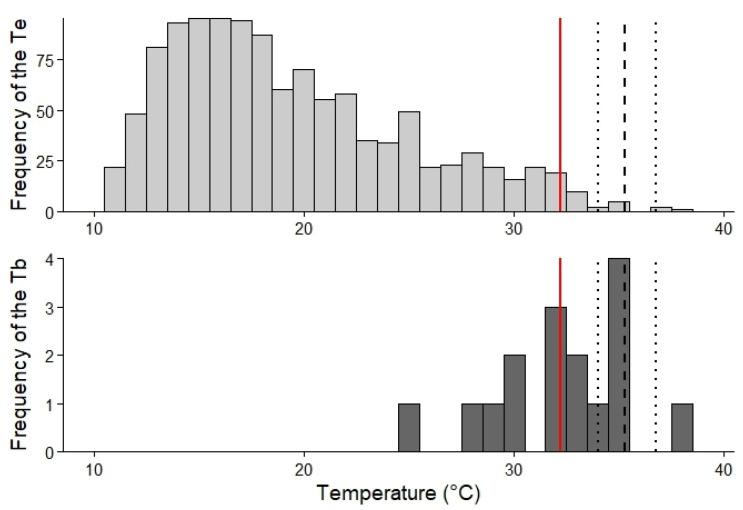
Frequency distributions of operative temperatures (*T_e_*; **upper panel**) and field body temperatures (*T_b_*; **lower panel**) of *Phymaturus williamsi*. The red solid line indicates mean *T_b_*, the black dashed line represents mean preferred temperature (*T_pref_*), and dotted lines show the setpoint temperature range. The comparison between environmental thermal availability (*T_e_*) and body temperatures (*T_b_*) illustrates the degree of thermoregulatory adjustment relative to the species’ preferred thermal range.

Most *T_b_* values (81.25%) were below the lower limit of the preferred temperature range (*T_set_*), whereas 12.5% fell within this range and 6.25% exceeded it (Figure 1). Thermoregulatory precision was *db* = 2.85, whereas habitat thermal quality was *de* = 16.82. Thermoregulatory effectiveness was high (*E* = 0.83), indicating that *T_b_* values were relatively close to *T_pref_* despite low environmental temperatures (mean *T_e_* = 18.05 ± 6.35 °C, *n* = 2735; Figure 1). For *P. williamsi*, 99.21% of operative temperature (*T_e_*) records were below the set-point range (Figure 1). Only 0.61% of *T_e_* values fell within this range, whereas 0.18% exceeded its upper boundary. Moreover, 97.57% of *T_e_* records were below the mean *T_b_*, and only 2.43% were equal to or higher than *T_b_* (Figure 1).

### 3.2. Effect of Temperature on Locomotor Performance

**Figure 2:**
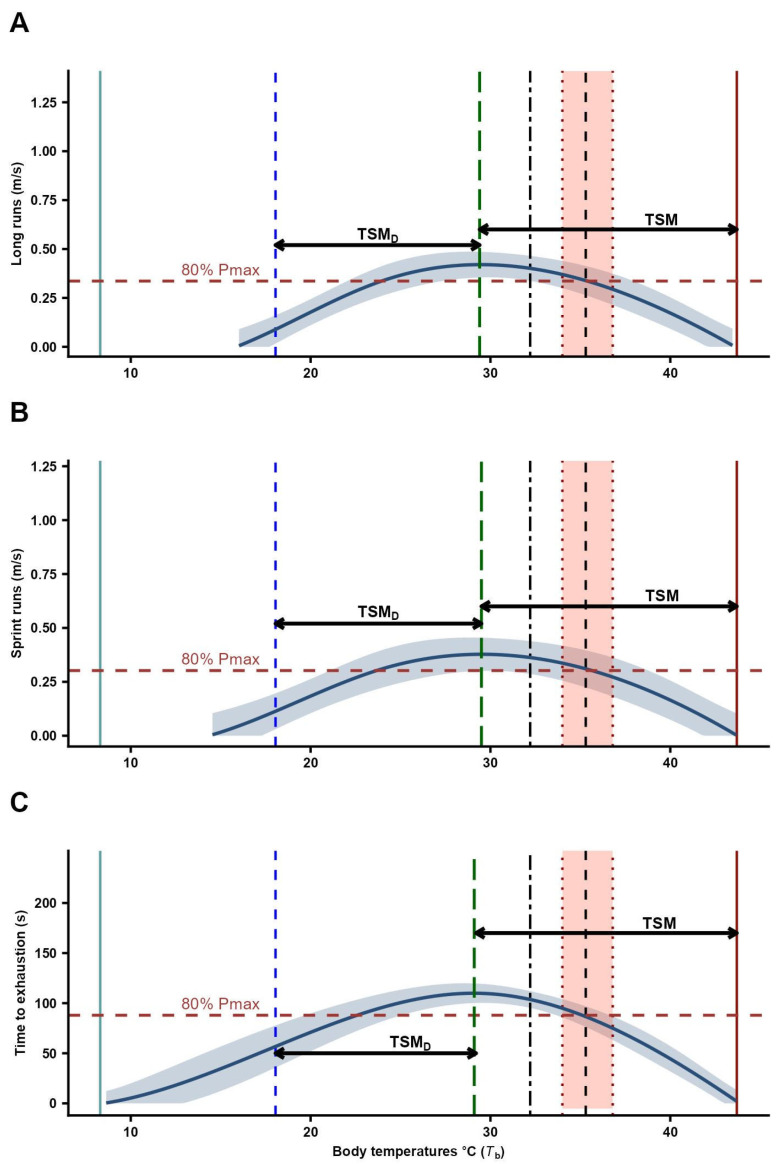
Thermal performance curves (TPC) describing the relationship between body temperature treatments and performance metrics in *Phymaturus williamsi*. (**A**) Long-run performance (LR, m s^−1^) at 18, 21, 27, 32, and 35 °C. (**B**) Sprint performance (SR, m s^−1^) at 18, 21, 27, 32, and 35 °C; (**C**) stamina (Time to exhaustion, s) at 27, 32, and 35 °C. Solid blue curves represent GAM-predicted performance, fitted using individual size as a covariate, with semi-transparent blue shaded bands indicating the 95% confidence intervals (CI) around the predictions. Green long-dashed vertical lines indicate optimal temperatures (LR: *T_o_* = 29.4 °C; SR: *T_o_* = 29.5 °C; Stamina: *T_o_* = 29.1 °C). Red dashed horizontal lines represent the performance threshold corresponding to 80% of maximum performance (*B*_80_). Salmon shaded areas and red dotted vertical lines denote the selected temperature range (*T_set_* = 34–36.8 °C), while black dashed vertical lines mark the mean selected temperature (*T_pref_* = 35.3 °C). Solid light-blue and dark-red vertical lines indicate the critical thermal minimum (*CT_min_* = 8.3 °C) and maximum (*CT_max_* = 43.7 °C), respectively. Blue dashed vertical lines represent the mean operative environmental temperature (*T_e_* = 18.05 °C). Black dash–dot vertical lines indicate the mean field body temperature (*T_b_* = 32.21 °C). Horizontal double-headed arrows illustrate the thermal safety margins: TSM (between *T_o_* and *CT_max_*) and TSM_D_ (between *T_e_* and *T_o_*).

Locomotor performance increased with temperature for both long runs (LR) and sprint runs (SR), reaching peak values at intermediate temperatures (27–32 °C) and declining at 35 °C (Figure 2). Significant differences among temperature treatments were detected for LR (ANOVA on ranks: F_4,60_ = 5.77, *p* < 0.01). Individuals at 27, 32, and 35 °C exhibited significantly higher speeds than those at 18 °C, which showed the lowest performance (Table 2; Tukey post hoc: *p* < 0.01). Performance at 21 °C did not differ from 18 °C but was significantly lower than at 27 and 32 °C. No significant differences were detected between 27 and 32 °C, which exhibited the highest performance values. A similar pattern was observed for SR (F_4,60_ = 3.31, *p* < 0.05). The highest speeds occurred at 27 and 32 °C, both significantly higher than at 18 and 21 °C (Table 2; Tukey: *p* < 0.05). Performance at 21 °C did not differ from 18 °C, whereas values at 35 °C overlapped statistically with the remaining treatments.

Effect sizes (Cohen’s d) revealed large biological effects for both sprint and long-run performance (Table S1, Supplementary Material). For long-run performance (Figure S1, Supplementary Material), the largest effects were observed between extreme temperatures: 18 °C vs. 27 °C (d = −0.94, *p* < 0.001) and 18 °C vs. 32 °C (d = −0.95, *p* < 0.001). Moderate to large effects were found for 21 °C vs. 27 °C (d = −0.73, *p* < 0.001) and 21 °C vs. 32 °C (d = −0.73, *p* < 0.001). For sprint performance (Figure S2, Supplementary Material), medium to large effects were observed for 18 °C vs. 27 °C (d = −0.68, *p* < 0.05) and 18 °C vs. 32 °C (d = −0.68, *p* < 0.01), with moderate effects for 21 °C vs. 27 °C (d = −0.53, *p* < 0.01) and 21 °C vs. 32 °C (d = −0.52, *p* < 0.05). In contrast to the running performance metrics, stamina showed no significant differences among temperature treatments (27 °C, 32 °C, and 35 °C; all *p* > 0.05; Figure S3, Supplementary Material). Effect sizes were correspondingly small to trivial: 27 °C vs. 32 °C (d = 0.31, *p* = 0.361), 27 °C vs. 35 °C (d = 0.25, *p* = 0.500), and 32 °C vs. 35 °C (d = −0.05, *p* = 0.976).

Values of optimal temperature (*T_o_*) and maximum performance (*V_max_*) are presented in Table 1. For LR, individual maximum speed (*V_max,i_*) was not significantly related to body temperature (linear regression: F_1,14_ = 4.72, *p* > 0.05) or body mass. However, *V_max,i_* showed a positive and significant relationship with SVL (multiple linear regression: t_13_ = 6.22, *p* < 0.05), while body mass was not significant. Similarly, for SR, *V_max,i_* was not significantly associated with *T_b_* (F_1,14_ = 1.74, *p* > 0.05) or body mass. In contrast, SVL showed a positive and significant relationship with *V_max,i_* (t_13_ = 4.05, *p* < 0.01).

Endurance capacity did not vary significantly among temperatures (repeated-measures ANOVA on ranks: F_2,30_ = 1.42, *p* > 0.05), although the highest mean value was observed at 35 °C (Table 2, Figure 2). Maximum endurance (Table 1) was not significantly related to body temperature (F_1,14_ = 0.31, *p* > 0.05), nor to SVL or body mass (multiple regression: *p* > 0.05).

### 3.3. Thermal Tolerance and Thermal Vulnerability Indices

Mean critical thermal minimum (*CT_min_*) was 8.25 ± 1.71 °C and mean critical thermal maximum (*CT_max_*) was 43.66 ± 0.71 °C, resulting in a thermal tolerance range of 35.41 °C.

The thermal safety margin (TSM_D_; sensu Deutsch et al. [2]) was 12.4 °C for LR, 12.5 °C for SR, and 13.2 °C for endurance. The thermal safety margin (TSM; sensu Gómez Alés et al. [32]) was 14.3 °C for LR, 14.2 °C for SR, and 14.6 °C for endurance. Warming tolerance (WT) for *P. williamsi* was 25.63 °C, indicating high thermal tolerance to warming.

## 4. Discussion

Consistent with the thermal coadaptation hypothesis, optimal temperatures (*T_ₒ_*) of fitness-related thermal performance curves (TPCs) are expected to correlate with preferred body temperature (*T_pref_*) [9,33,34,35]. Under this framework, *T_o_* values of fitness-related TPCs are expected to correlate with *T_pref_*. However, this prediction was not supported in *P. williamsi*, as the estimated *T_o_* values for the different performance traits were consistently lower than the observed *T_pref_* (Table 2). Therefore, in terms of locomotor performance, our results do not support the thermal coadaptation hypothesis in this species. It is important to note, however, that our sample size was relatively small (*n* = 16), which may limit the statistical power to detect subtle associations among thermal traits. Therefore, non-significant relationships should be interpreted with caution.

Alternatively, the preferred body temperature (*T_pref_*) of *P. williamsi* may be under selection to optimize other physiological processes, such as reproduction, digestion, or growth [35,89,90]. This interpretation is supported by the observation that this species is able to reach maximal locomotor performance at body temperatures commonly experienced during its daily activity period (Table 2). In addition, field body temperatures (*T_b_*) were higher than the mean operative environmental temperature (*T_e_* = 18.05 °C), indicating that despite a limited availability of thermally suitable microhabitats, individuals can behaviorally thermoregulate to attain temperatures close to their performance optima. However, body temperatures near or above the optimal temperature (*T_o_*) for a given physiological process generally result in sharper declines in performance compared to temperatures below *T_o_* [3,26,28,34]. In this context, the high thermoregulatory effectiveness of *P. williamsi* may help buffer this trade-off between environmental temperatures and the thermal optima associated with locomotor performance [28]. In this context, the high thermoregulatory effectiveness of *P. williamsi* may help buffer this trade-off between environmental temperatures and the thermal optima associated with locomotor performance [28]. It is important to note that the variables compared here derive from different contexts: *T_b_* and *T_e_* reflect field conditions integrating environmental heterogeneity and behavioral thermoregulation, whereas *T_pref_* and *T_o_* are obtained under controlled laboratory conditions. As a result, direct comparisons among these variables should be interpreted with caution, as mismatches may arise not only from biological processes but also from differences in measurement context.

Environmental constraints imposed by the topography of Puna and high-Andean environments [57,66,91] may explain the mismatch observed between *T_b_* and *T_pref_* in *P. williamsi*. Although Gómez Alés et al. [29] suggested that local features of the study area—such as the north–south orientation of the valley and intermediate vegetation cover—reduce wind exposure and its cooling effects, other factors may still limit the attainment of higher body temperatures. For instance, variation in solar radiation and the shading produced by slopes can expose individuals to either higher or lower temperatures, respectively, thereby constraining *T_b_* and reducing opportunities for effective thermoregulation [26,52,79,92,93]. Consistent with this, a previous study conducted in the same area reported low thermal quality of the environment for *P. williamsi* at Quebrada La Puerta [57]. In our study, nearly 100% of operative temperature (*T_e_*) records fell below the set-point range (*T_set_*), while more than 80% of *T_b_* values were also below this range. These results highlight the strong thermal constraints imposed by the environment, which likely limit the ability of *P. williamsi* to attain body temperatures close to its preferred temperature.

Despite the low operative temperatures recorded, *P. williamsi* attained relatively high body temperatures, likely as a result of its high thermoregulatory effectiveness (*E* = 0.83), thus supporting Prediction 2 of this study. However, our results differ from those previously reported for the same population (*E* = 0.50; Laspiur et al. [57]). These discrepancies may be explained by methodological differences between studies. On the one hand, we used PVC models to estimate operative temperatures, whereas Laspiur et al. [57] employed copper models. On the other hand, the thermoregulatory effectiveness (E) estimated in this study is based solely on data collected during October–November (spring), while the value reported by Laspiur et al. [57] integrates records from October (spring), December (summer), and April (autumn). These methodological differences may obscure the effects of seasonal thermal variation characteristic of the Puna region [57,66,75,79,91,94], as well as potential differences in heat transfer properties between the materials used in the biophysical models to estimate *T_e_*. Therefore, caution is needed when comparing thermoregulatory indices across studies that differ in temporal scale and methodological approaches. In this sense, our results should be interpreted within the seasonal context in which data were collected, as thermal biology and performance traits in Andean lizards may vary across seasons [71,95,96]. Future studies incorporating broader temporal sampling would help to evaluate the consistency of these patterns throughout the annual cycle.

Locomotor performance in sprint trials increased with temperature up to an optimal value and declined thereafter (Figure 2). This pattern, characterized by relatively high performance across a broad range of temperatures, has been reported as an adaptive trait in several lizard species inhabiting cold and temperate environments of the Andes and Patagonia [6,18,27,28,29,32,55,97,98,99,100]. The ability to maintain high locomotor performance over a wide thermal range has been primarily linked to habitat characteristics and ecological demands, including predator escape, social interactions, and foraging [32,100]. In contrast, endurance capacity has been associated with diet and microhabitat use [32,101]. In this context, the herbivorous diet of *P. williamsi* suggests that high endurance capacity may not be essential, as individuals likely move short distances between food resources within rocky habitats. This interpretation is consistent with the relatively small home range reported for the species [19,102]. This combination of broad thermal performance and reduced reliance on endurance may reflect an ecological strategy prioritizing short, rapid movements over sustained activity.

In contrast to previous studies in liolaemid lizards [32,97,98,103], we found a positive relationship between *V_max_* for both locomotor modes and SVL, whereas body mass showed no significant effect. This pattern suggests that locomotor performance in *P. williamsi* may be more closely associated with linear body dimensions than with overall body mass, potentially reflecting biomechanical factors such as stride length or limb proportions. While this could be related to biomechanical factors such as stride length or limb proportions, our data do not allow us to directly test these mechanisms. Similarly, variation in muscle composition may also influence performance, but this was not evaluated in the present study [104,105]. Therefore, these interpretations should be considered with caution. Alternatively, the observed pattern may be consistent with functional and ecological factors associated with escape behavior. In this context, predation pressure from birds [106], together with the documented escape responses of this species [107], could favor traits related to rapid acceleration and sprint performance. Interestingly, previous work has suggested that a weak or absent relationship between body size and speed may reduce trade-offs between energy acquisition and escape capacity [32]. In this sense, *P. williamsi* may represent a useful model to further explore the links between locomotor performance, morphology, and its herbivorous lifestyle. Notably, maximum locomotor performance showed limited sensitivity to temperature, suggesting that this species may maintain relatively stable sprint capacity across a range of ecologically relevant body temperatures. Additionally, although no significant differences between sexes have been reported for some thermal traits in *Phymaturus* [57,66], sexual variation has been documented in other liolaemid lizards [72,75,95]. Therefore, the potential influence of sex on locomotor and thermal traits cannot be ruled out and should be explicitly addressed in future studies with larger sample sizes.

Contrary to our Prediction 3, *P. williamsi* exhibited relatively high values of thermal vulnerability indices (Figure 2), suggesting that the species may be able to buffer the projected increases in environmental temperature (~2–3.5 °C) for the Central Andes region [27,59,108]. In particular, the relatively high TSM_D_ values indicate that current operative temperatures are below the thermal optima for locomotor performance, suggesting that environmental warming could initially bring body temperatures closer to optimal conditions. Similarly, the high TSM values reflect a substantial distance between optimal performance temperatures and critical thermal maxima, indicating a broad physiological buffer before reaching upper thermal limits. In addition, the high WT values suggest that environmental temperatures remain far from lethal thresholds, reinforcing the idea of a relatively low immediate risk from direct thermal stress. In general, lizards inhabiting temperate and cold environments of the Andes and Patagonia tend to behave as thermal generalists and exhibit relatively high thermal safety margins and warming tolerance, consistent with our estimates [6,18,26,27,28,29,32,51,57,66,97,99,100,109,110]. However, these relatively high tolerance values should not be interpreted as an absence of vulnerability. Even moderate increases in environmental temperature could alter the availability and temporal distribution of suitable operative temperatures, potentially constraining activity times and increasing exposure to suboptimal or stressful thermal conditions [3,4,30]. In highly seasonal and thermally variable environments such as the Andean highlands, small shifts in temperature regimes may also disrupt the balance between thermoregulation, foraging, and predator avoidance [29,40,107]. Moreover, because *P. williamsi* relies on specific microhabitats (e.g., rocky substrates and crevices) for thermoregulation [19], its capacity to buffer climate warming will likely depend not only on physiological tolerance but also on the availability of thermal refuges. Thus, habitat structure and microhabitat heterogeneity may play a critical role in mediating the effects of climate change on this species.

Taken together, our results suggest that the vulnerability of *P. williamsi* to climate warming may not be primarily driven by its physiological limits, but rather by ecological constraints on the availability of suitable thermal conditions. In this sense, climate change may impact this species indirectly by altering the thermal landscape and restricting opportunities for effective thermoregulation and activity.

## 5. Conclusions

In conclusion, *P. williamsi* is a eurythermic species that exhibits thermal sensitivity in locomotor performance and behaves as an effective thermoregulator. Despite showing an ecophysiological capacity to tolerate the moderate warming projected for the Central Andes [2,80,108,111], its persistence may ultimately depend on the availability of suitable microhabitats that buffer environmental temperature extremes. This study contributes to improving our understanding of the thermal ecophysiology and climate vulnerability of this species, complementing previous findings [57] and expanding current knowledge on its thermal tolerance and locomotor performance. Nevertheless, future studies should incorporate seasonal variation and microhabitat thermal heterogeneity to more comprehensively assess its persistence in the Puna region of the Andean highlands.

## Figures and Tables

**Table 1:** Mean values (±standard deviation) of morphological, thermal, and locomotor performance traits for *Phymaturus williamsi* (*n* = 16). Variables include snout–vent length (SVL), body mass (BM), preferred body temperature (*T_pref_*), and the lower (*T_setLo_*) and upper (*T_setUp_*) limits of the set-point range. Locomotor performance metrics are presented separately for sprint runs (SR), long runs (LR), and endurance, including optimal temperature for performance (*T_o_*), thermal performance breadth (*B*_80_) maximum speed (*V_max_*) for SR and LR, and maximum endurance capacity (s).

Variable	Mean ± SD	Range
SVL (mm)	100.06 ± 3.87	—
BM (g)	35.09 ± 5.20	—
*T_pref_* (°C)	35.28 ± 1.76	—
*T_setLo_* (°C)	34.01 ± 3.13	—
*T_setUp_* (°C)	36.78 ± 1.09	—
*T_o_* (SR) (°C)	29.50 ± 3.26	—
*B*_80_ (SR) (°C)	—	23.67–35.64
*V_max_* (SR) (m s^−1^)	0.37 ± 0.08	—
*T_o_* (LR) (°C)	29.40 ± 3.11	—
*B*_80_ (LR) (°C)	—	23.95–35.39
*V_max_* (LR) (m s^−1^)	0.42 ± 0.09	—
*T_o_* (Endurance) (°C)	29.10 ± 3.35	—
*B*_80_ (Endurance) (°C)	—	22.59–35.10
Endurance	109.92 ± 34.56	—

**Table 2:** Mean values (±standard deviation) of sprint runs (SR, long-run speed (LR), and endurance capacity of *Phymaturus williamsi* (*n* = 16) across experimental temperature treatments. Sprint and long-run speeds are expressed in m s^−1^, and endurance as time to exhaustion (s). Endurance trials were conducted only at 27, 32, and 35 °C.

Traits	Temperature Treatments				
	18 °C	21 °C	27 °C	32 °C	35 °C
Sprint runs	0.09 ± 0.07	0.17 ± 0.06	0.42 ± 0.07	0.40 ± 0.06	0.28 ± 0.06
Long runs	0.05 ± 0.05	0.16 ± 0.05	0.47 ± 0.05	0.45 ± 0.05	0.29 ± 0.05
Endurance	-	-	110.2 ± 8.26	95.90 ± 7.77	98.00 ± 8.25

## Data Availability

The raw data supporting the conclusions of this article will be made available by the authors on request.

## Supplementary Materials

The following supporting information can be downloaded at: https://www.mdpi.com/article/10.3390/biology15090729/s1. Figure S1: Effect sizes (Cohen’s d) for pairwise temperature comparisons in sprint performance (SR) of *Phymaturus williamsi*. Bars represent standardized differences between temperature treatments, with colors indicating statistical significance: dark blue (*p* < 0.05) and gray (non-significant). Values above bars are Cohen’s d coefficients. Dashed reference lines indicate interpretation of thresholds: d = 0.2 (small effect), d = 0.5 (medium effect), and d = 0.8 (large effect). Negative d values indicate higher performance at the warmer temperature of each pair. The largest effects were observed between extreme temperatures (18 °C vs. 27 °C and 18 °C vs. 32 °C: d = −0.68), representing medium-to-large biological effects. Horizontal bar plot orientation facilitates comparison of effect magnitudes across temperature pairs. Figure S2: Effect sizes (Cohen’s d) for pairwise temperature comparisons in long-run performance (LR) of *Phymaturus williamsi*. Bars represent standardized differences between temperature treatments, with colors indicating statistical significance: dark blue (*p* < 0.05) and gray (non-significant). Values above bars are Cohen’s d coefficients. Dashed reference lines indicate interpretation of thresholds: d = 0.2 (small effect), d = 0.5 (medium effect), and d = 0.8 (large effect). Negative d values indicate higher performance at the warmer temperature of each pair. The largest effects were observed between extreme temperatures (18 °C vs. 27 °C: d = −0.94; 18 °C vs. 32 °C: d = −0.95), representing large biological effects. Horizontal bar plot orientation facilitates comparison of effect magnitudes across temperature pairs. Figure S3: Effect sizes (Cohen’s d) for pairwise temperature comparisons in stamina (time to exhaustion, seconds) of *Phymaturus williamsi*. Bars represent standardized differences between temperature treatments, with colors indicating statistical significance: dark blue (*p* < 0.05) and gray (non-significant). Values above bars are Cohen’s d coefficients. Dashed reference lines indicate interpretation of thresholds: d = 0.2 (small effect), d = 0.5 (medium effect), and d = 0.8 (large effect) following Cohen [87]. All comparisons were non-significant (*p* > 0.05), with effect sizes ranging from small to trivial (d = 0.31 for 27 °C vs. 32 °C; d = 0.25 for 27 °C vs. 35 °C; d = −0.05 for 32 °C vs. 35 °C). This indicates that stamina is less sensitive to temperature variation within the tested range (27–35 °C) compared to sprint and long-run performance. Table S1: Effect sizes (Cohen’s d) for pairwise temperature comparisons in sprint performance (SR), long-run performance (LR), and stamina of *Phymaturus williamsi*.
