# Supplementary material for: Thermophysiology and Locomotor Performance of the Andean Lizard Phymaturus williamsi: Vulnerable to Rising Temperatures?
Source: Biology (Basel). 2026 May 5;15(9):729. doi: 10.3390/biology15090729 (PMC13162878; doi:10.3390/biology15090729)
Supplement: Supplementary file 1 [file biology-15-00729-s001.zip › biology-4246252-supplementary.pdf]

**Supplementary Material**

To complement null hypothesis significance testing, we calculated standardized effect sizes (Cohen's d) for all pairwise temperature comparisons following Lakens (2013). Cohen's d expresses the magnitude of the difference between two temperature treatments in standard deviation units, allowing interpretation of biological significance beyond statistical significance. Following Cohen (1988) conventions, we interpret d = 0.2 as a small effect, d = 0.5 as a medium effect, and d = 0.8 as a large effect. Positive d values indicate higher performance at the warmer temperature, while negative values indicate higher performance at the cooler temperature. For repeated-measures designs, we approximated d using the formula $d = t \times \sqrt{(2/n)}$, where t is the t-ratio from the post hoc Tukey test and n is the sample size (Lakens 2013).

Table S1. Effect sizes (Cohen's d) for pairwise temperature comparisons in sprint performance (SR), long-run performance (LR), and stamina of *Phymaturus williamsi*.

| Metric | Comparison | Difference | Cohen's d | Interpretation | p-value |
|---|---|---|---|---|---|
| LR | 18 vs 27 | -0.422 | -0.94 | Large | <0.001 |
| LR | 18 vs 32 | -0.401 | -0.95 | Large | <0.001 |
| LR | 18 vs 35 | -0.239 | -0.57 | Medium | 0.009 |
| LR | 21 vs 27 | -0.316 | -0.73 | Medium–Large | <0.001 |
| LR | 21 vs 32 | -0.295 | -0.73 | Medium–Large | <0.001 |
| LR | 21 vs 35 | -0.133 | -0.33 | Small | 0.280 |
| LR | 27 vs 35 | 0.183 | 0.42 | Small–Medium | 0.088 |
| LR | 32 vs 35 | 0.162 | 0.40 | Small | 0.120 |
| LR | 18 vs 21 | -0.106 | -0.25 | Small | 0.542 |
| LR | 27 vs 32 | 0.021 | 0.05 | Trivial | 0.998 |
| SR | 18 vs 27 | -0.334 | -0.68 | Medium–Large | 0.001 |
| SR | 18 vs 32 | -0.316 | -0.68 | Medium–Large | 0.001 |
| SR | 18 vs 35 | -0.198 | -0.43 | Small–Medium | 0.082 |
| SR | 21 vs 27 | -0.250 | -0.53 | Medium | 0.017 |
| SR | 21 vs 32 | -0.232 | -0.52 | Medium | 0.020 |
| SR | 21 vs 35 | -0.114 | -0.26 | Small | 0.526 |
| SR | 27 vs 35 | 0.136 | 0.29 | Small | 0.409 |
| SR | 32 vs 35 | 0.118 | 0.26 | Small | 0.493 |
| SR | 18 vs 21 | -0.084 | -0.18 | Trivial | 0.802 |
| SR | 27 vs 32 | 0.019 | 0.04 | Trivial | 0.999 |
| Stamina | 27 vs 32 | 14.315 | 0.31 | Small | 0.361 |
| Stamina | 27 vs 35 | 12.147 | 0.25 | Small | 0.500 |
| Stamina | 32 vs 35 | -2.167 | -0.05 | Trivial | 0.976 |

Note: Negative d values indicate higher performance at the warmer temperature of the pair. Interpretation thresholds: d = 0.2 (small), d = 0.5 (medium), and d = 0.8 (large) following Cohen (1988).

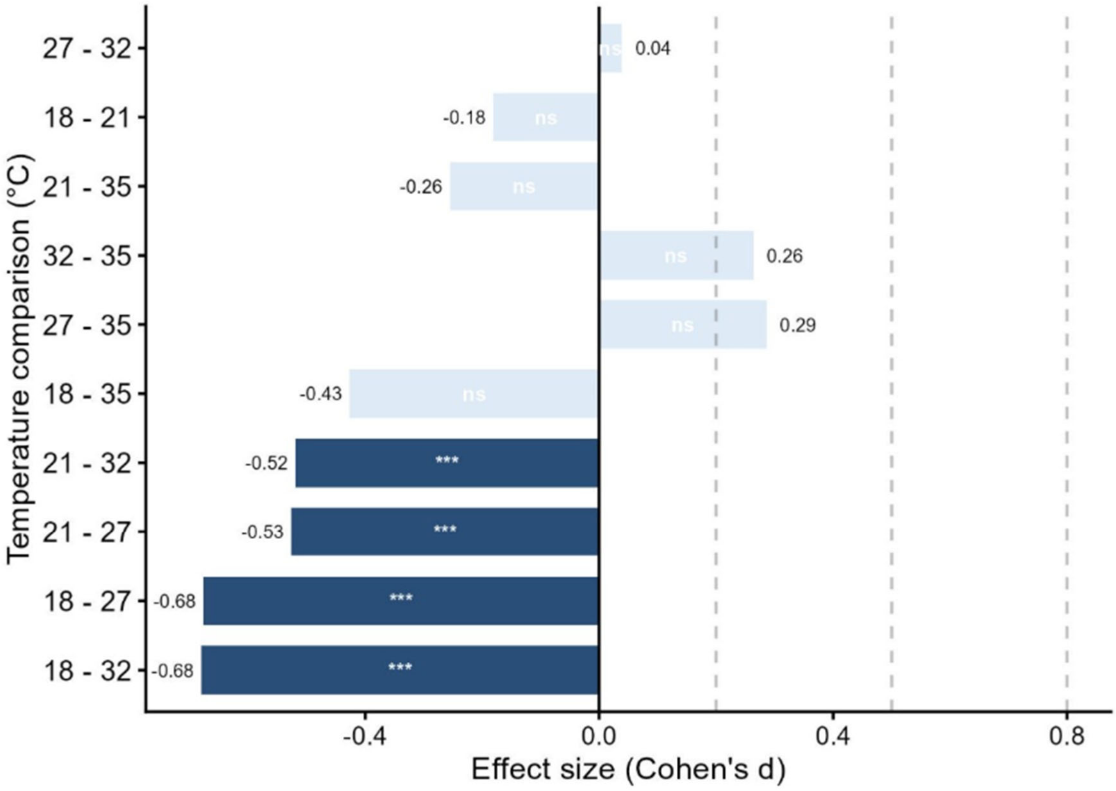


Figure S1. Effect sizes (Cohen's d) for pairwise temperature comparisons in sprint performance (SR) of *Phymaturus williamsi*. Bars represent standardized differences between temperature treatments, with colors indicating statistical significance: dark blue ($p < 0.05$) and gray (non-significant). Values above bars are Cohen's d coefficients. Dashed reference lines indicate interpretation of thresholds: d = 0.2 (small effect), d = 0.5 (medium effect), and d = 0.8 (large effect). Negative d values indicate higher performance at the warmer temperature of each pair. The largest effects were observed between extreme temperatures (18°C vs. 27°C and 18°C vs. 32°C: d = -0.68), representing medium-to-large biological effects. Horizontal bar plot orientation facilitates comparison of effect magnitudes across temperature pairs.

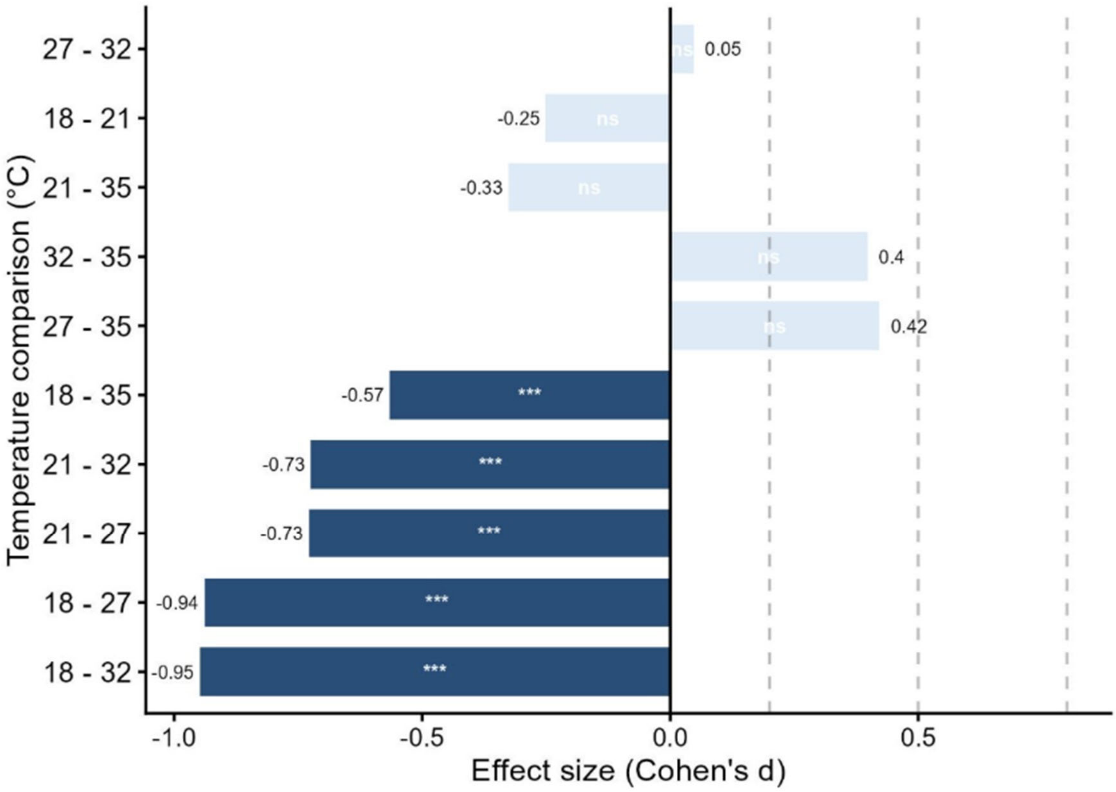


Figure S2. Effect sizes (Cohen's d) for pairwise temperature comparisons in long-run performance (LR) of *Phymaturus williamsi*. Bars represent standardized differences between temperature treatments, with colors indicating statistical significance: dark blue ($p < 0.05$) and gray (non-significant). Values above bars are Cohen's d coefficients. Dashed reference lines indicate interpretation of thresholds: d = 0.2 (small effect), d = 0.5 (medium effect), and d = 0.8 (large effect). Negative d values indicate higher performance at the warmer temperature of each pair. The largest effects were observed between extreme temperatures (18°C vs. 27°C: d = -0.94; 18°C vs. 32°C: d = -0.95), representing large biological effects. Horizontal bar plot orientation facilitates comparison of effect magnitudes across temperature pairs.

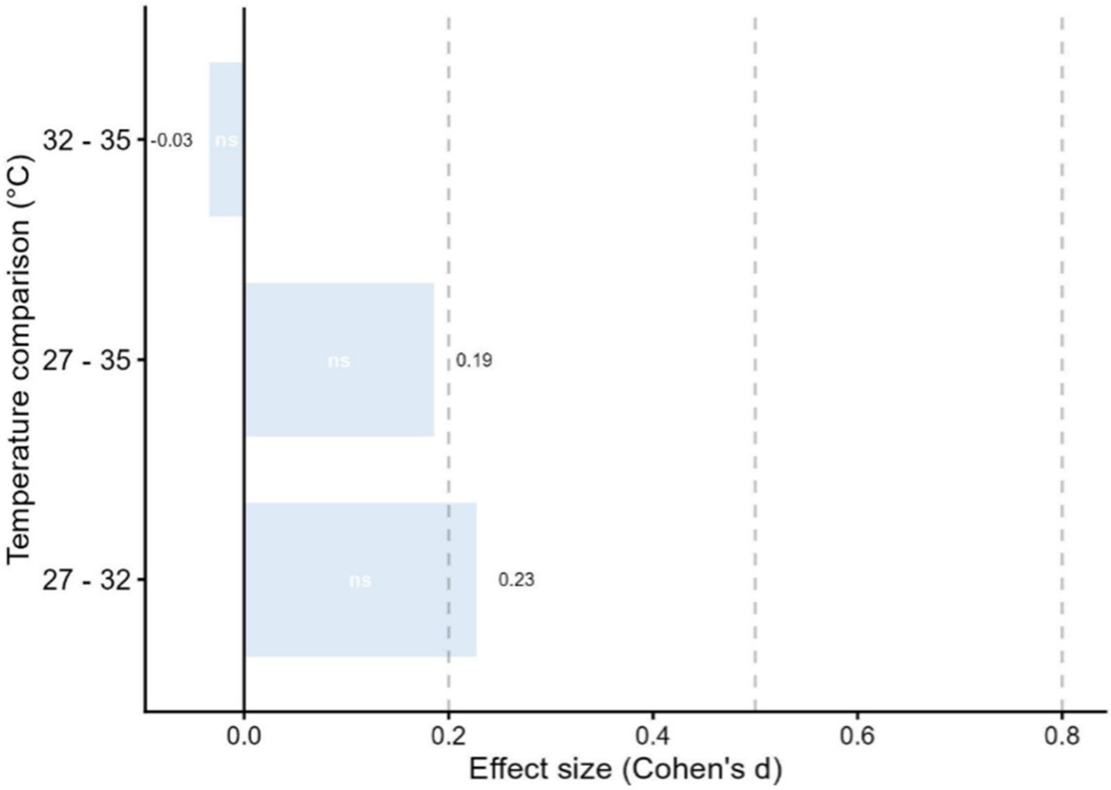


Figure S3. Effect sizes (Cohen's d) for pairwise temperature comparisons in stamina (time to exhaustion, seconds) of *Phymaturus williamsi*. Bars represent standardized differences between temperature treatments, with colors indicating statistical significance: dark blue ($p < 0.05$) and gray (non-significant). Values above bars are Cohen's d coefficients. Dashed reference lines indicate interpretation of thresholds: $d = 0.2$ (small effect), $d = 0.5$ (medium effect), and $d = 0.8$ (large effect) following Cohen (1988). All comparisons were non-significant ($p > 0.05$), with effect sizes ranging from small to trivial ($d = 0.31$ for 27°C vs. 32°C; $d = 0.25$ for 27°C vs. 35°C; $d = -0.05$ for 32°C vs. 35°C). This indicates that stamina is less sensitive to temperature variation within the tested range (27–35°C) compared to sprint and long-run performance.
